# Method for Deodorizing Iodoform

**Published:** 1887-10

**Authors:** Will Taft


### A Method for Deodorizing Iodoform.

Of all the various substances used for the purpose of correcting the disgusting odor of iodoform there is nothing more effective than the aroma of a good quality of coffee such as Java or Mocha, the latter preferably as being the most pungent.

The method I have adopted for this purpose is as follows: Take a half ounce vial and thoroughly dry it by subjecting it to heat. Then obtain one or the other of the above brands of coffee, pulverized if possible, otherwise pulverize it yourself, then fill the vial about one-third full, without packing, fill the rest of the space with iodoform then keep it tightly corked. In following the above directions you will have one of the most valuable of remedies relieved of the most potent objection to its use. It will of course be understood that where larger quantities are to be used a larger amount of coffee will be necessary for deodorization.

WILL TAFT.

